# Distinct patterns of natural selection in Na^+^/H^+^ antiporter genes in *Populus euphratica* and *Populus pruinosa*

**DOI:** 10.1002/ece3.2639

**Published:** 2016-12-08

**Authors:** Yuxia Wu, Kuibin Meng, Xiaohui Liang

**Affiliations:** ^1^ State Key Laboratory of Grassland Agro‐EcosystemSchool of Life SciencesLanzhou UniversityLanzhouGansuChina

**Keywords:** Na^+^/H^+^ antiporter, natural selection, nucleotide diversity, *Populus euphratica*, *Populus pruinosa*

## Abstract

Salt tolerance genes constitute an important class of loci in plant genomes. Little is known about the extent to which natural selection in saline environments has acted upon these loci, and what types of nucleotide diversity such selection has given rise to. Here, we surveyed genetic diversity in three types of Na^+^/H^+^ antiporter gene (*SOS*,* NhaD,* and *NHX*, belonging to the cation/proton antiporter 1 family), which have well**‐**characterized essential roles in plant salt tolerance. Ten Na^+^/H^+^ antiporter genes and 16 neutral loci randomly selected as controls were sequenced from 17 accessions of two closely related members of the genus *Populus*,* Populus euphratica* and *Populus pruinosa*, section *Turanga*, which are native to northwest China. The results show that salt tolerance genes are common targets of natural selection in *P. euphratica* and *P. pruinosa. *Moreover, the patterns of nucleotide variation across the three types of Na^+^/H^+^ antiporter gene are distinctly different in these two closely related *Populus* species, and gene flow from *P. pruinosa* to *P. euphratica* is highly restricted. Our results suggest that natural selection played an important role in shaping the current distinct patterns of Na^+^/H^+^ antiporter genes, resulting in adaptive evolution in *P. euphratica* and *P. pruinosa*.

## 1 Introduction

The ability of an organism to undergo biological adaptation to the environment is a product of long‐term evolution (Darwin, 1859). A population is able to evolve when it contains individuals with heritable variation in traits facilitating functional diversification and adaptation. Adaptive evolution has been discussed in detail with respect to genes involved in human diseases and/or pathogen response pathways (Manjurano et al., 2015; Tishkoff et al., 2001; Vigano et al., 2012). In plant genomes, genes related to salt adaptation constitute an important group of loci. It is still largely unknown whether differences in mutation rates across the genome, consistent with natural selection in response to environmental stresses, can account for the evolution of salt tolerance‐related genes in plants. Positive selection will decrease nucleotide diversity in proximity to loci under selective pressure and increase the prevalence of low‐frequency SNPs, whereas balancing selection or local adaptation increases both nucleotide diversity and the prevalence of medium‐frequency SNPs (Biswas & Akey, 2006; Fu & Li, 1993; Morrell, Lundy, & Clegg, 2003; Tajima, 1989). Although the effects of demographic history on nucleotide diversity and site frequency distributions can mimic the effects of selection, demographic influences affect the whole genome while selection targets specific loci. Thus, demographic effects can be estimated using a genomewide set of reference genes (Akey et al., 2004; Glinka et al., 2003; Wright & Gaut, 2005). Multilocus scans that detect outliers from neutral expectations are more apt to successfully identify plant genes influenced by natural selection, as, for example, was carried out in studies on drought and/or salt tolerance‐related genes in sunflower, wild tomato, and *Pinus pinaster* (Eveno et al., 2008; Fischer et al., 2011; Kane & Rieseberg, 2007), “phenology genes” in balsam poplar (*Populus balsamifera*, Keller et al., 2011, 2012), and candidate genes for cold hardiness in coastal Douglas fir (*Pseudotsuga menziesii* var. *menziesii*, Eckert et al., 2009).

Recent advances in genetic analysis and the advent of the genomic era have permitted the isolation and identification of genes responsible for salt tolerance pathways. Salt resistance is a quantitative character controlled by multiple genes in plants, with a given plant species typically containing hundreds or thousands of salt‐responsive genes (Brosche et al., 2005; Gong et al., 2005; Ma et al., 2013; Qiu et al., 2011; Sottosanto, Gelli, & Blumwald, 2004; Yang et al., 2013). Na^+^/H^+^ antiporters provide one mechanism for the removal of sodium from the cytoplasm in order to maintain low cytoplasmic sodium concentrations in plant cells (Bassil, Ohto, et al., 2011; Bassil, Tajima, et al., 2011; Fukuda et al., 2004; Shi et al., 2003; Yokoi et al., 2002). Plant Na^+^/H^+^ antiporters belong to the monovalent cation/proton antiporter 1 (CPA1) superfamily (Brett, Donowitz, & Rao, 2005). At least three types of Na^+^/H^+^ antiporter, *NHX*,* NhaD,* and *SOS* **,** which differ in subcellular location**,** have been found in plants (Barrero‐Gil, Rodriguez‐Navarro, & Benito, 2007; Blumwald & Poole, 1985; Shi et al., 2000), and it has been suggested that they play important roles in coping with increased Na^+^ influx and in compartmentalizing Na^+^ within subcellular compartments under salt stress (Bassil, Ohto, et al., 2011; Bassil, Tajima, et al., 2011; Fukuda et al., 2004; Gaxiola et al., 1999; Shi et al., 2003; Yamaguchi et al., 2003). Plant salt tolerance can be enhanced by over‐expression of Na^+^/H^+^ antiporters (Apse et al., 1999; Shi et al., 2003), and the expression profiles of these genes differ between closely related salt‐sensitive and salt‐tolerant species (Kant et al., 2006; Zahrana et al., 2007).

**Figure 1:**
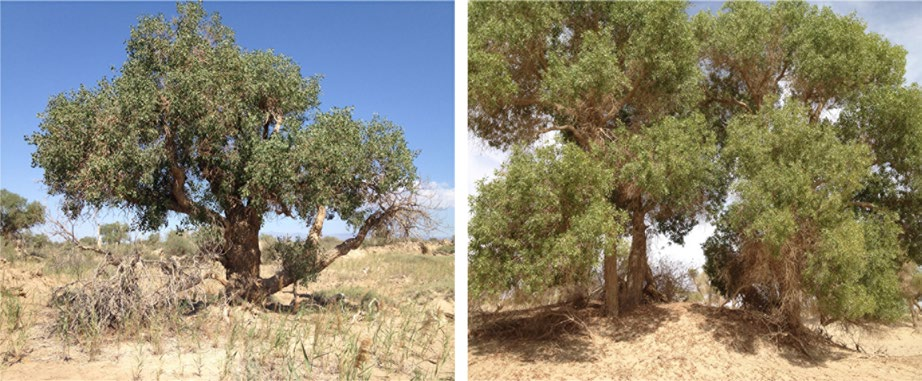
*Populus euphratica* Olivier (left) and *Populus pruinosa* Schrenk (right), distributed in desert area in northwest China. Photographs by Yuxia Wu

The sister species *Populus euphratica* Olivier and *Populus pruinosa* Schrenk are members of the *Populus* section *Turanga* Bunge; they grow in semi‐arid regions of China and are known for their high levels of stress tolerance (Figure 1). Both species play important roles in the arid ecosystems of northwest China (Li et al., 2003, 2006). Surveys of DNA sequence variability in salt tolerance genes from closely related *Populus* species distributed in similar high‐salinity environments may contribute to a comprehensive understanding of the genetic basis of salt adaptation of *Populus* and the other plants. For this study, we selected three types of Na^+^/H^+^ antiporter gene which were identified in previous studies as being affected by salt stress in *P. euphratica* (Hu & Wu, 1987; Ottow et al., 2005; Wu et al., 2007; Ye et al., 2009), and sequenced almost the complete gene coding regions for ten Na^+^/H^+^ antiporter genes in these three classes, and 16 neutral loci randomly selected as controls**,** in order to analyze nucleotide diversity in these two *Populus* species.

## 2 Materials and Methods

### 2.1 Plant materials

**Figure 2:**
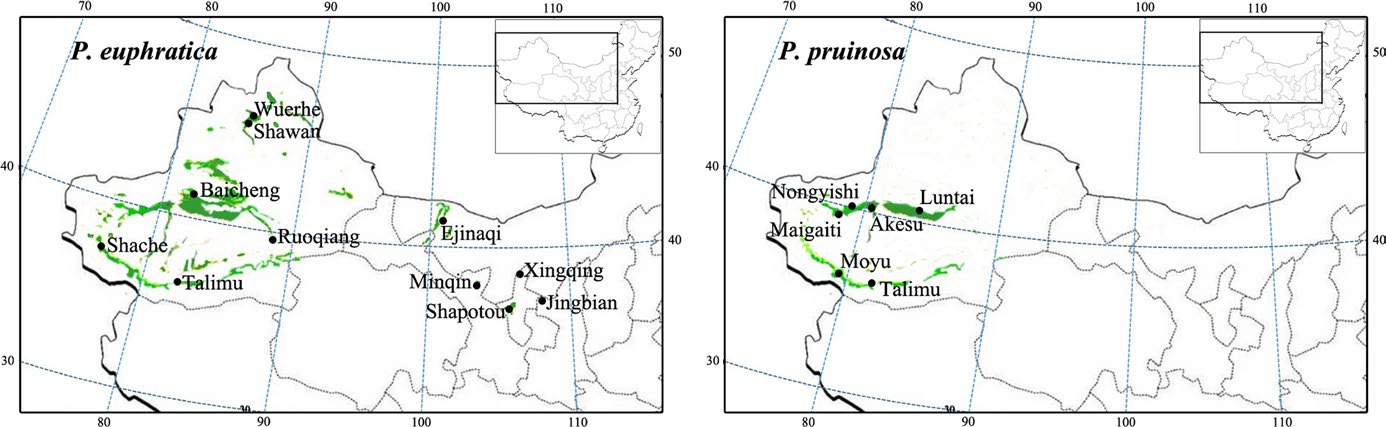
Locations of populations sampled and accessions of *Populus euphratica* and *Populus pruinosa* used in this study. The geographic distribution ranges of *P. euphratica* and *P. pruinosa* in China are shown in green

Leaf tissues from 17 different populations, six of *P. pruinosa* and eleven of *P. euphratica*, were collected from sites covering the full geographic range of each species in northwest China (Figure 2). For both species, ten to fifteen individuals were sampled from each population; the sampled individuals were separated by at least 100 m to minimize the potential for sampling the same clonal individual. Four to eight individuals per population were selected randomly for this study. DNA was extracted from silica gel‐dried leaves by a modified CTAB method (Doyle & Doyle, 1987).

### 2.2 Amplification, cloning, and sequencing

For this work, we selected three types of Na^+^/H^+^ antiporter gene on the basis of previous studies that have identified genes affected by salt stress in *P. euphratica* (Hu & Wu, 1987; Ottow et al., 2005; Wu et al., 2007; Ye et al., 2009). *SOS1* and *SOS1B* encode plasma membrane‐localized transporters, the six *NHX* genes belong to the vacuolar type Na^+^/H^+^ antiporter group, and the two *NhaD* genes are chloroplast‐localized (Barrero‐Gil et al., 2007; Blumwald & Poole, 1985; Shi et al., 2000). We constructed primers (Table S1) for these Na/H antiporter genes using data from the poplar genome database (http://genome.jgi-psf.org/Poptr1/Poptr1.home.html), making the assumption that the gene family consisted of the ten members previously described (Barrero‐Gil et al., 2007; Blumwald & Poole, 1985; Shi et al., 2000).

The sequenced regions of the Na^+^/H^+^ antiporter loci ranged from 3,890 to 8,858 bases in length and contained complete coding regions (excluding some poor‐quality sequences in the case of *SOS1B*) for each gene (Figure S1), including a total of 17,812 bp of coding region and 32,463 bp of noncoding region, resulting in a total length of 50,325 bp and 49,518 bp in *P. euphratica* and *P. pruinosa*, respectively, across all genes. The total numbers of sequenced nonsynonymous, synonymous, and noncoding sites for all the Na^+^/H^+^ antiporter genes are given in Table S2. We directly sequenced most of the genes from PCR products after treatment with ExoSAP‐IT (USB Corp., Cleveland, OH, USA). Several portions of the genes contained long fragments with segregating indels that prevented direct sequencing. In these cases, PCR products were cloned into the pGEM19‐T vector (Takara, China) after preparing the DNA using the recommended protocol for the AxyPrep DNA Gel Extraction kit (AXYGEN, China), and three to five clones were sequenced. The 16 reference loci (Table S3) were randomly selected from a set used to develop estimates of nucleotide diversity in *P. balsamifera* (Olson et al., 2010). We PCR‐amplified and directly sequenced these reference loci from all 76 sampled individuals (Table S4). The 16 reference loci contained 162–639 bp of coding sequence and ranged in length from 415 to 687 bp per gene, with a total length of 9,761 bp. Aligned sequences were edited manually using Aligner v.5.1.0 (Codon Code Corporation, Dedham, MA), with all posterior probabilities >.8 and all polymorphic and heterozygous sites visually confirmed. For the ten Na^+^/H^+^ antiporter genes and 16 reference loci, exon and intron boundaries were determined from cDNA sequences obtained from the GenBank database or genomic sequences (Ma et al., 2013; Tuskan et al., 2006).

### 2.3 Data analysis

To resolve haplotypes from sequences obtained directly from PCR products, phase v. 2.1 (Stephens & Scheet, 2005; Stephens, Smith, & Donnelly, 2001) was used. After excluding insertions/deletions (indels), genetic parameters were estimated using DnaSP v.5.10 (Librado & Rozas, 2009). We implemented the IMa2 (Hey, 2010, 2004; Hey & Nielsen, 2014) program using a Markov Chain Monte Carlo (MCMC) approach to analyze gene flow between the two species. Posterior probability densities (proportional to likelihoods) of the model parameters were used to assess significance (Guo et al., 2013). Arlequin 3.1 (Excoffier, Laval, & Schneider, 2005) was used to calculate population structure statistics (*F*_ST_). The statistical significance of *F*_ST_ for each locus was calculated by comparing the observed values with the distribution of *F*_ST_ calculated from 10,000 permutations of sequences among populations.

The influence of selection on individual salt tolerance genes was tested using multiple methods. For each species, we separately generated 10^5^ simulated data sets under the neutral model of Hudson, Kreitman, and Aguade (2005) to simulate a genomewide pattern of diversity using diversity estimates from our 16 reference loci. These data sets were used to assess the probability that diversity estimates for the salt tolerance genes were consistent with neutral expectations. The simulated data sets consisted of 80 and 50 chromosomes in *P. euphratica* and *P. pruinosa*, respectively, numbers which reflected average sample sizes across loci for each species (http://home.uchicago.edu/rhudson1/source/mksamples.html). Absolute nucleotide differentiation for each salt tolerance gene was calculated as π_T‐S_ = π_T_ ‐ π_S_, where π_T_ is the total nucleotide diversity using all the samples from the two species and π_S_ is the average nucleotide diversity within each of the species (Charlesworth, 1998; Keller et al., 2012). Statistical significance was determined by comparing the observed π_T_ ‐π_S_ to the 95% confidence interval calculated from the variation among 10^4^ neutral coalescent simulations for each salt tolerance gene using DnaSP v.5.10 (Librado & Rozas, 2009), and these estimates were based on the number of segregating sites and assumed no recombination. The HKA test (Hudson et al., 2005) was applied to evaluate the ratio of silent polymorphisms within species to the divergence between species across multiple loci. We conducted an HKA test (Hudson et al., 2005) using the multilocus HKA program available from Jody Hey (https://bio.cst.temple.edu/~hey/software/software.htm) to assess whether differences could be identified across all loci with 10^4^ simulations. We then used maximum‐likelihood HKA (mlHKA) to conduct statistical tests comparing each salt tolerance locus between species (Wright & Charlesworth, 2004; http://wright.eeb.utoronto.ca/programs/). To compare the model in which selection is hypothesized and the neutral model at specific loci, the divergence time parameter (T) was set at 15 (Ma et al., 2013; Tuskan et al., 2006) in the mlHKA program with 10^5^ MCMC cycles. Comparisons were performed by carrying out a likelihood‐ratio test using chi‐squared values to determine statistical significance. The population selection parameters (γ = 2NeS, where Ne is the effective population size and S is the selection parameter) were determined using the mkprf software described in Bustamante et al. (2002). We defined sites in the salt tolerance genes and the 16 reference loci as replacement or silent sites, depending on whether or not each polymorphism altered the amino acid at a given site in *P. euphratica* and/or *P. pruinosa* relative to the amino acid present at the same site in *Populus trichocarpa*. mkprf was based on the posterior distribution of the sample parameters using the Monte Carlo Markov Chain (MCMC) approach. We ran the simulation for 10^7^ cycles and, after discarding the first 10^4^ as “burn‐in,” we sampled every 10th iteration. We summarized the selection parameters for each salt tolerance gene and reference locus using the mean and 95% distribution confidence intervals.

## 3 Results

### 3.1 Nucleotide diversity and population differentiation

Ten Na^+^/H^+^ antiporter genes were sequenced from a total of 76 individuals: 52 individuals from eleven *P. euphratica* populations and 24 individuals from six *P. pruinosa* populations (Figure 2). After excluding indels, the aligned Na^+^/H^+^ antiporter sequence for each locus ranged in length from 3,890 bp (*NHX5*) to 8,858 bp (*SOS1*) (Figure S1 and Table S2). Statistics on sequence polymorphism for each locus are presented in Table S2.

**Figure 3:**
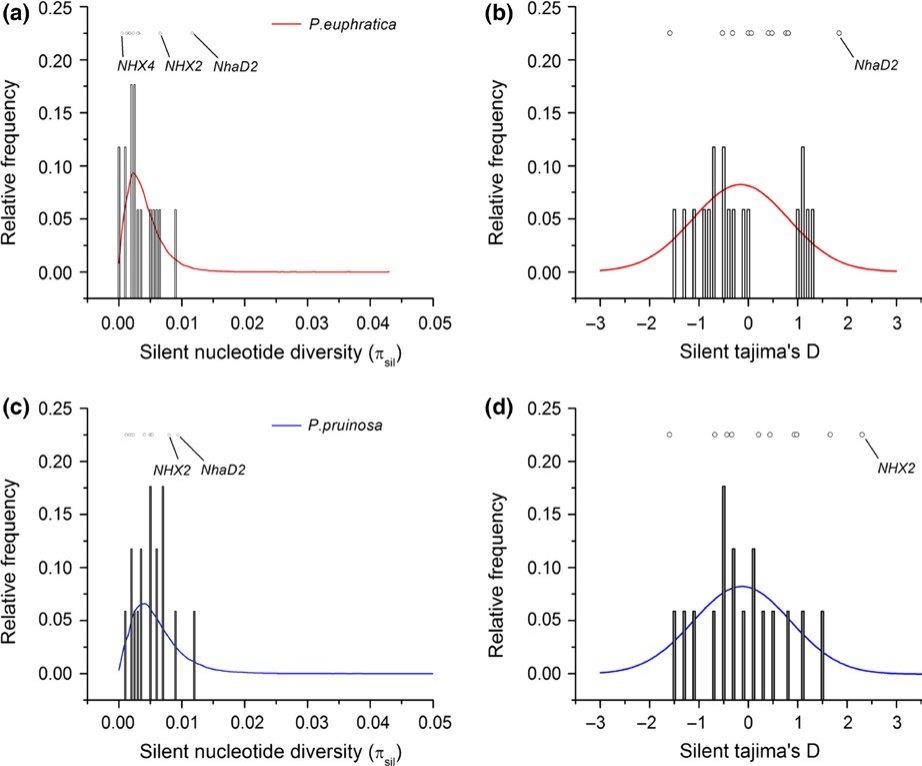
Comparisons of nucleotide diversity and spectrum of site frequencies at silent nucleotide substitution sites for salt tolerance genes (open circles and points) and reference loci (open and shaded bars) in *Populus euphratica* (a and b) and *Populus pruinosa* (c and d) and coalescent simulations of a standard neutral model for *P. euphratica* (red) and *P. pruinosa* (blue)

Most salt tolerance genes and all reference loci had higher nucleotide diversity in *P. pruinosa* than in *P. euphratica* (Tables S2 and S4). The average nucleotide diversity across all salt tolerance‐related genes was higher in *P. pruinosa* (θ_w_ = 0.0030 ± 0.00097) than in *P. euphratica* (θ_w_ = 0.0025 ± 0.00076; Table S2). The mean synonymous nucleotide diversity (π_sil_) across the 16 reference loci was 0.0034 for *P. euphratica* and 0.0053 for *P. pruinosa* (Figure 3 and Table S4).

The 16 reference loci exhibited differentiation among *P. euphratica* populations (mean *F*_ST_ = 0.257, range = 0–0.424) but significantly less differentiation among *P. pruinosa* populations (mean *F*_ST_ = 0.149, range = 0.024–0.381, Table S4). The *F*_ST_ values for salt tolerance genes among populations within species varied between 0.253 and 0.545 in *P. euphratica* and between 0.124 and 0.463 in *P. pruinosa* (Table S2).

### 3.2 Gene flow and introgression

**Figure 4:**
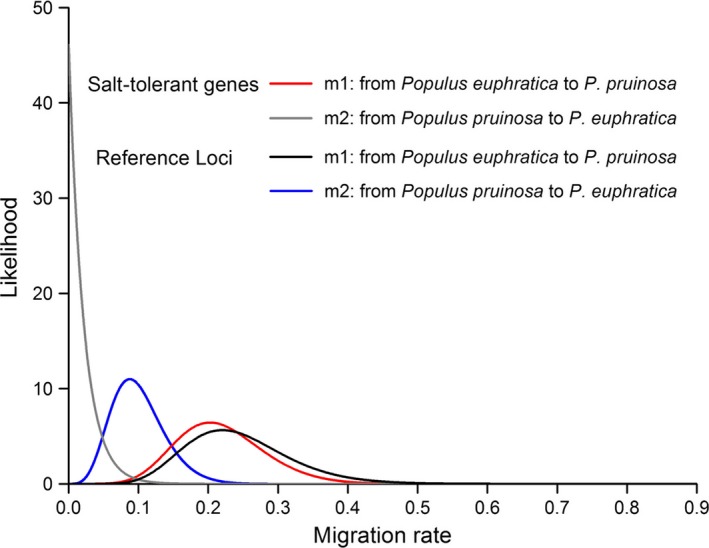
Likelihood distributions for migration rate estimates under the IMa2 model

Gene flow between *P. euphratica* and *P. pruinosa* was examined using the IMa2 model, and the marginal posterior density distribution for migration rates is shown in Figure 4. Estimates of migration parameters were nonzero for both species at reference loci, with m1 = 0.219 (from *P. euphratica* to *P. pruinosa*) and m2 = 0.087 (from *P. pruinosa* to *P. euphratica*); the probabilities of the migration rate for both direction were strongly statistically significant (*p *<* *.001). For the salt tolerance genes, the migration rate was m1 = 0.203 from *P. euphratica* to *P. pruinosa* whereas the migration rate in the opposite direction was almost zero (m2 = 0.0003). Thus, salt tolerance gene flow from *P. euphratica* to *P. pruinosa* was greater; gene flow from the opposite direction was very restricted.

### 3.3 Site frequency distributions and diversity vs. divergence

**Figure 5:**
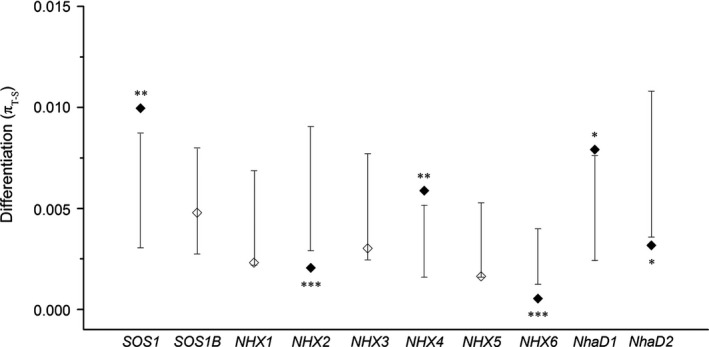
Coalescent analysis of nucleotide differentiation between *Populus euphratica* and *Populus pruinosa*. Shaded bars denote 95% confidence intervals around the simulated distribution from the neutral model. Diamonds are observed values of differentiation, with observed values falling outside of the simulated distributions depicted by solid diamonds (*p <* *.05, **p <* *.01, ***p <* *.001)

**Figure 6:**
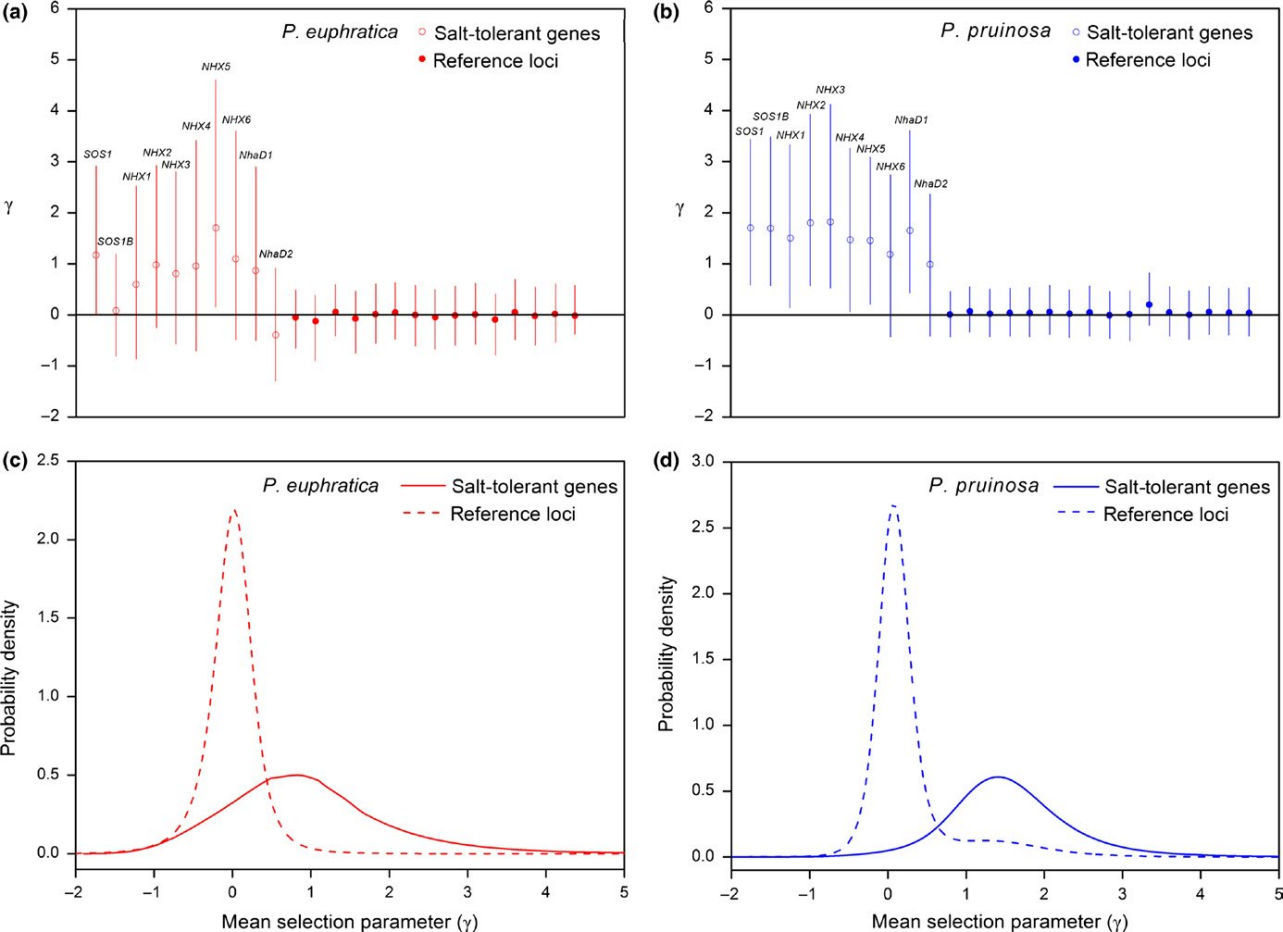
Means of the posterior distributions of the selection parameters (γ = 2N_e_s) from a hierarchical Bayesian mkprf analysis. Selection parameters for Na^+^/H^+^ antiporter salt tolerance genes (red and blue open circles) and reference loci (red and blue points) are shown; error bars are 95% confidence intervals (red and blue vertical lines) for *Populus euphratica* (a) and *Populus pruinosa* (b). Estimated distribution of the mean value of the selection parameter (γ) across salt tolerance genes (red solid line) and reference loci (red dashed line) in *P. euphratica* (c) and across salt tolerance genes (blue solid line) and reference loci (blue dashed line) in *P. pruinosa* (d)

Some salt tolerance genes in both *P. euphratica* and *P. pruinosa* exhibited silent site diversity (π_sil_) that was inconsistent with neutral expectations (Figure 3). *NHX4*,* NHX2,* and *NhaD2* in *P. euphratica* (Figure 3a,b), *NHX2*, and *NhaD2* in *P. pruinosa* (Figure 3c,d) exhibited outlier π_sil_ and D_sil_ values compared to neutral expectations. HKA tests comparing diversity within both species to divergence from *P. trichocarpa* (Tuskan et al., 2006) showed that salt tolerance genes had significant differences from a neutral model in *P. euphratica* (x^2^ = 23.88, *df* = 9, *p* < .01), and near‐significant differences in *P. pruinosa* (x^2^ = 16.21, *df* = 9, *p* = .06; Table 1). mlHKA tests were performed to assess whether diversity and divergence for salt tolerance loci were consistent with neutral evolution from *P. trichocarpa* (Table 2). In *P. euphratica*,* NHX2* and *NhaD2* had elevated diversity compared to divergence, whereas *NHX4* and *SOS1* showed decreased diversity. In *P. pruinosa*,* NhaD1* exhibited significant deviation from neutrality, and *NhaD2* exhibited near‐significant deviation from neutrality (*p* = .065, *k* = 1.85; Table 2). At the whole‐gene level, as measured by π_T‐S_, more than half of the salt tolerance genes showed significant differentiation (*SOS1*,* NHX2*,* NHX4*,* NHX6*,* NhaD1,* and *NhaD2*) or near‐significant differentiation (*NHX1* and *NHX5*) in excess of the expectations for neutral simulations between *P. euphratica* and *P. pruinosa* (Figure 5). We performed mkprf analysis for the ten salt tolerance genes in this study and the 16 randomly selected neutral reference loci, using *P. trichocarpa* **,** for which sequence data are publicly available**,** as the outgroup. We divided the loci that we analyzed into two classes (salt tolerance genes and reference loci) and obtained the posterior distribution parameters shown in Figure 6. The results showed that the salt tolerance genes have different patterns of replacement site polymorphism relative to the reference loci in both species (Figure 6). *NhaD2* had a significantly negative selection parameter, whereas the other salt tolerance genes (with the exception of *SOS1B*) had significantly positive selection parameters, in *P. euphratica* (Figure 6a). Salt tolerance genes had significantly positive selection parameters in *P. pruinosa* (Figure 6b). The estimated selection parameters**,** with mean values of 0.89 and 1.49 in *P. euphratica* and *P. pruinosa*, respectively, were significantly greater than zero in both species (Figure 6c,d). However, none of the reference loci showed a significantly negative or positive selection parameter distribution in either species (Figure 6). We thus found evidence for predominantly beneficial gene substitutions in salt tolerance genes**,** but not in reference loci**,** in both species.

**Table 1:** HKA test results for 10 salt tolerance genes in *P. euphratica* and *P. pruinosa*

Species	Deviation^a^	*df* b	*p*_chi_ c
*P. euphratica*	23.8829	9	.00792**
*P. pruinosa*	16.2086	9	.06265

^a^Sum of deviations in the chi‐square test.

^b^Degrees of freedom.

^c^Chi‐square distribution probability.

*p < .05, **p < .01.

**Table 2:** mlHKA test results for salt tolerance genes in *Populus euphratica* and *Populus pruinosa*

Species	Description	ln L^a^	Likelihood‐ratio statistics	*p* b	*K* c
*P. euphratica*	Neutral (all *k* = 1)	−90.5620			
	*SOS1*	−87.9450	5.2340	.022*	0.43
	*SOS1B*	−90.6341	−0.1442	#NUM	1.15
	*NHX1*	−90.3898	0.3444	.557	1.27
	*NHX2*	−88.5555	4.0130	.045*	1.78
	*NHX3*	−90.0357	1.0526	.305	1.46
	*NHX4*	−87.7454	5.6332	.018*	0.35
	*NHX5*	−90.7698	−0.4156	#NUM	1.10
	*NHX6*	−91.4150	−1.7060	#NUM	0.67
	*NhaD1*	−90.6519	−0.1798	#NUM	1.04
	*NhaD2*	−87.8897	5.3446	.021*	2.04
*P. pruinosa*	Neutral (all *k* = 1)	−83.8881			
	*SOS1*	−83.6798	0.4166	.519	0.75
	*SOS1B*	−83.0617	1.6528	.199	0.59
	*NHX1*	−83.7073	0.3616	.548	1.24
	*NHX2*	−83.6987	0.3788	.538	1.31
	*NHX3*	−82.6406	2.4950	.114	1.63
	*NHX4*	−83.8798	0.0166	.897	1.07
	*NHX5*	−83.6745	0.4272	.513	1.27
	*NHX6*	−84.2675	−0.7588	#NUM	1.06
	*NhaD1*	−79.5194	8.7374	.003**	0.32
	*NhaD2*	−82.1915	3.3932	.065	1.86

^a^The likelihood value of the model.

^b^The possibility of the chi‐square distribution.

^c^The selection parameter for the gene.

*p < .05, **p < .01.

## 4 Discussion

### 4.1 Salt tolerance genes are common targets of natural selection in *P. euphratica* and *P. pruinosa*

The genes responsible for salt tolerance have been extensively studied in many plants, especially in the model plant *Arabidopsis thaliana* (Apse et al., 1999; Bassil, Ohto, et al., 2011; Bassil, Tajima, et al., 2011; Qiu et al., 2002; Shi et al., 2000; Yamaguchi et al., 2003, 2005). An analysis of nucleotide polymorphism and divergence in the salt tolerance‐related genes in 20 ecotypes of *A. thaliana* provided little evidence for any recent effect of positive selection (Puerma & Montserrat, 2013). In the present study, several genes related to salt tolerance appear to have been recent targets of positive selection in *P. euphratica* and *P. pruinosa* according to the results of the mkprf analyses. However, most salt tolerance genes in both *P. euphratica* and *P. pruinosa* exhibited silent site diversity (π_sil_) which were consistent with neutral expectations, the exceptions being *NhaD2* in *P. euphratica* and *NHX2 in P. pruinosa*. A positive value for the selection parameter γ indicated an excess of nonsynonymous substitutions in nine of the 10 Na^+^/H^+^ antiporter genes in *P. euphratica* and eight of the 10 Na^+^/H^+^ antiporter genes in *P. pruinosa*. An excess of nonsynonymous substitutions is a strong indicator of positive selection**,** which has been used to detect**,** for example**,** sex‐biased adaptive evolution in *Drosophila melanogaster* (Proschel, Zhang, & Parsch, 2006) and disease response genes in loblolly pine (Ersoz et al., 2010). Meanwhile, HKA tests showed significant differences from a neutral model in *P. euphratica* (*p* < .01), and near‐significant differences in *P. pruinosa* (*p* = .06) for the 10 Na^+^/H^+^ antiporter genes*. *A comparison of nucleotide polymorphisms and divergence indicates that salt tolerance genes are more frequently targets of natural selection than are reference loci in *P. euphratica* and *P. pruinosa*. In northwest China, natural populations of *P. euphratica* and *P. pruinosa* are distributed only in desert regions, where there is little precipitation and higher salt habitats occur, and conditions are consequently hostile to plants. Comparative studies have indicated that these species can withstand salt stress better than any other poplar species tested**,** on the basis of growth, photosynthetic performance, and survival under salt stress (Chen et al., 2003; Hukin et al., 2008; Wang et al., 2007). Our results showed that salt tolerance genes have undergone greater divergence between the two species than the 16 reference loci, indicating that adaptive evolution of these genes has taken place in both *P. euphratica* and *P. pruinosa*.

### 4.2 Genetic signatures indicate differences in selective pressure acting on salt tolerance genes between *P. euphratica* and *P. pruinosa*

Population genomic signatures of selection can include decreased polymorphism within populations, elevated differentiation among populations (*F*_ST_), and linkage disequilibrium resulting in neutral expectations failing to be met (Beaumont, 2005; Keller et al., 2012; Tang, Thornton, & Stoneking, 2007). The mean synonymous nucleotide diversity values across the 16 regions we sampled in *P. euphratica* (π_sil_ = 0.0033) and *P. pruinosa* (π_sil_ = 0.0053) largely fall within the ranges found across other *Populus* species based on estimates from multiple loci, *P. trichocarpa* (π_sil_ = 0.0029, Gilchrist et al., 2006), *P. balsamifera* (π_sil_ = 0.0045, Olson et al. 2010), and *P. tremula* (π_sil_ = 0.0125, Ingvarsson, 2008). Mean nucleotide diversity across the ten salt tolerance genes in *P. euphratica* (θ_w_ = 0.0025) and *P. pruinosa* indicated that the latter had much higher nucleotide diversity, with an average value of θ_w_ = 0.0030. When we compared the 16 reference loci between these two species, nucleotide diversity was still lower in *P. euphratica* (θ_w_ = 0.0025) than in *P. pruinosa* (θ_w_ = 0.0034). These results are consistent with our recent study of CpDNA in these two species, which indicated that total gene diversity (H_T_) in *P. euphratica* is H_T_ = 0.447, lower than the *P. pruinosa* value of H_T_ = 0.590 (Wang et al., 2011). These results are also consistent with those of another of our recent studies, which were inferred from six *NHX* type Na^+^/H^+^ antiporter gene fragments (Guo et al., 2013) and other candidate nuclear loci (Wang et al., 2014) from these two species. Variation at particular loci resulting from different environmental selection pressures will accentuate levels of population differentiation and thus result in higher *F*_ST_ values (Biswas & Akey, 2006; Keller et al., 2012). Our previous study using the same set of populations showed that demographic effects were not as a cause of the haplotype structure in *P. euphratica* (Wang et al., 2014). Of the two species, salt tolerance genes showed a higher level of genetic differentiation among populations in *P. euphratica* (*F*_ST_ = 0.407) than in *P. pruinosa* (*F*_ST_ = 0.223). In addition, most salt tolerance genes exhibited genetic signatures indicating different degrees of selection, relative to the neutral model, between *P. euphratica* and *P. pruinosa*. Although results from our previous study showed that these two desert poplar species underwent speciation about 0.66–1.37 million years ago, during a period when deserts were widespread and glaciations oscillations were occurring frequently in central Asia (Wang et al., 2014), the lower nucleotide diversity and higher *F*_ST_ value in *P. euphratica* compared with *P. pruinosa* is striking, and most salt tolerance genes show significantly different genetic signatures, revealing that natural selection has been acting differentially on these sibling species.

### 4.3 Countervailing selection acting on gene flow from *P. pruinosa* to *P. euphratica*

Complicating factors, such as gene flow, mutation, natural selection, and adaptation, provide the impetus for the gradual process of evolution (Lenormand, 2002; Morjan & Rieseberg, 2004). Gene flow is considered to delay the progress of speciation by homogenizing the genetic components and making the mechanisms that give rise to new species more complex (Lenormand, 2002; Slatkin, 1987). As *P. euphratica* has a wider geographic distribution than that of *P. pruinosa*, the lower nucleotide diversity in the former compared to the latter species is somewhat surprising. *P. pruinosa* has an earlier flowering time making the pollen flow from *P. euphratica* to *P. pruinosa* unrealistic (Guo et al., 2013). One possible factor to be considered is unbalanced gene flow between the two species, for the 16 reference loci, with more introgression taking place from *P. euphratica* to *P. pruinosa*. We found that gene flow between *P. pruinosa* and *P. euphratica* was asymmetric. These results were consistent with our previous study using SSR markers and other neutral genes (Guo et al., 2013; Wang et al., 2011, 2014). Interestingly, our findings showed that gene flow from *P. pruinosa* to *P. euphratica* for salt tolerance genes was completely blocked. Antagonism between gene flow and natural selection has been found in the case of the alleles of the gene encoding the bA subunit in yellow‐billed pintails living at different altitudes (McCracken et al., 2009). Observations from completed whole‐genome sequences for plants have shown that a high proportion of diversity has resulted from lineage‐specific adaptive evolution (Ma et al., 2013; Tuskan et al., 2006)**.** Our results indicated that salt tolerance alleles that are transferred from *P. pruinosa* to *P. euphratica* may confer lower fitness and thus be quickly eliminated from *P. euphratica*, resulting in gene flow being completely prevented; in the meantime, gene flow is still occurring at unlinked reference loci. Our results showed that the gene introgression of salt tolerance genes from *P. pruinosa* into *P. euphratica* has been eliminated, implying that the natural selection has been one of the evolutionary forces driving the divergence of these two *Populus* species.

Na^+^/H^+^ antiporters are essential for cellular salt and pH homeostasis throughout the plant kingdom. We detected distinct patterns of natural selection in salt tolerance genes, patterns which may have favored population fitness in saline regions where *P. euphratica* was located. These different patterns may have not only triggered speciation between *P. euphratica* and *P. pruinosa*, but also helped the two species to maintain their genetic distinctness and respective geographic distributions. It will therefore be informative to determine whether the genetic differentiation between these two *Populus* species, in particular with respect to the traits underlying their adaptation to extreme environments, is due to variation in a small number of key genes with large effects or to more complex genetic variation.

## Conflict of Interest

None declared.

## Data Accessibility

The sequences of each locus reported here were deposited in GenBank under accession numbers KX132138–KX132807 and KX156957–KX158184.
