# Supplementary material for: Distinct patterns of natural selection in Na+/H+ antiporter genes in Populus euphratica and Populus pruinosa
Source: Ecol Evol. 2016 Dec 8;7(1):82–91. doi: 10.1002/ece3.2639 (PMC5214168; doi:10.1002/ece3.2639)
Supplement: Supplementary file 1 [file ECE3-7-82-s001.doc]

**Supporting Information for online publication**

**
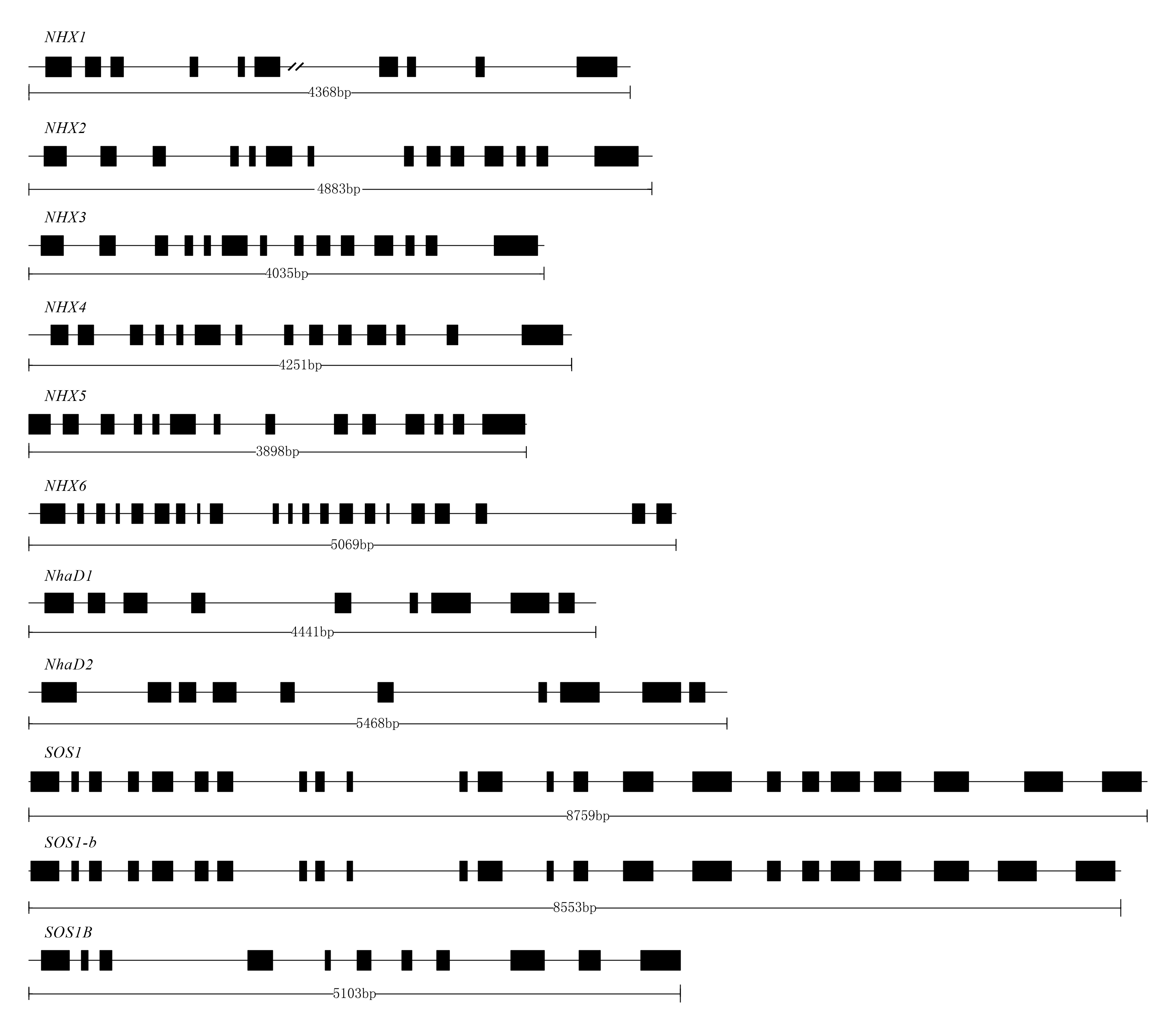
**

**Fig. S1** Locations and sizes of the sequenced regions of salt tolerance genes; the shaded boxes correspond to translated regions; the lines connecting the boxes are introns; the sequenced regions and sizes are shown under the boxes.

**Table S1** Gene-specific primers used for PCR and sequencing of 10 salt tolerance

| Gene | forward primer name |  | forward primer sequence(5ˊ-3ˊ) | | reverse primer name | |  | reverse primer sequence(5ˊ-3ˊ) |
| --- | --- | --- | --- | --- | --- | --- | --- | --- |
| *NHX1* | nhx1-1F |  | GAGAAGGAGTCTTTCTAGGTTCTCA |  | | nhx1-1R |  | ATAACCAAAATAAACCACAAACATC |
|  | nhx1-2F |  | CTTTGCCTGAAATCAAGTAGTGGTG |  | | nhx1-2R |  | GCTTCCCATGTGATTCAAGATGTAT |
|  | nhx1-3F |  | AGCCCGCTTGCTTAATCCCTA |  | | nhx1-3R |  | CAAAGCACATGCTATTTGCATGAGC |
|  | nhx1-4F |  | ATCTTCATGCTTTACCCATTTTGCC |  | | nhx1-4R |  | ATTCCCAGCATGTAAAGGATC |
|  | nhx1-5F |  | GCTGGTCTTTGGCAATACAAT |  | | nhx1-5R |  | TTTACCATCACGCCCACTCAT |
| *NHX2* | nhx2-1F |  | TTGTTTTTGCTGCTTTGAATGCAGG |  | | nhx2-1R |  | CTCTGTATGAACACTGGGTAG |
|  | nhx2-2F |  | CACTCAAACTACCCAGTGTTCATAC |  | | nhx2-2R |  | AACTACACATAAATACATGCGTGAT |
|  | nhx2-3F |  | TTGATGCCCAGTCCATACAAA |  | | nhx2-3R |  | AAACCAAGTCAAATGCCAGAC |
|  | nhx2-4F |  | GCGTTCAACCTCATCCCTCCT |  | | nhx2-4R |  | GATGGAAAAAGAAGCTGTCAG |
|  | nhx2-5F |  | GAATAGCAGAGCAAATTGTGTAGGC |  | | nhx2-5R |  | CTTCTGAACAGGAATGCCAAA |
| *NHX3* | nhx3-1F |  | TCCTTAGATGGACAGTACAGAGACG |  | | nhx3-1R |  | ATAATCAAAGGAGCACTGGGGCAAC |
|  | nhx3-2F |  | TATGCCTAGTCACCATGCTCC |  | | nhx3-2R |  | GCTAACTCAATCCCATCTACC |
|  | nhx3-3F |  | TGTGCCGATTCAATTAAACAC |  | | nhx3-3R |  | TCAAATGCCAGATGTCAGGAT |
|  | nhx3-4F |  | TCATCAATTCATTCAACCTCATCCC |  | | nhx3-4R |  | ACAGTCCGTTGACTCAGAGCTAAAC |
|  | nhx3-5F |  | ACCCTCTTAACATCTTCTATCC |  | | nhx3-5R |  | CAGGGCTTCATTATTCTCACA |
| *NHX4* | nhx4-1F |  | GGCTTCTCGCTGTCACTCTA |  | | nhx4-1R |  | CTTCACCAGGAGTCTCATACC |
|  | nhx4-2F |  | ACAATACTTTGTCTTGTTAGTGCTG |  | | nhx4-2R |  | ATTGAGAGAAATCTTACCTCGG |
|  | nhx4-3F |  | GCTCTACTTCGGCAGGTTAGT |  | | nhx4-3R |  | CTGATGTTCCAGGGCTGA |
|  | nhx4-4F |  | TTGTAAGTGATAGGTGAGGCT |  | | nhx4-4R |  | GCCTGACCTGGTGAACTG |
|  | nhx4-5F |  | CAATCTGTTCTGTGATGATAGG |  | | nhx4-5R |  | GGCACCTCCCCACTCTGAAATC |
| *NHX5* | nhx5-1F |  | ATCTCGAATGCTGCTATGTTTGGCC |  | | nhx5-1R |  | CCGATAGCTGCAGATGCAGGAAGCG |
|  | nhx5-2F |  | TGCGAGGAAGGATTTGGT |  | | nhx5-2R |  | AGCTGCTACCGCTAGGCG |
|  | nhx5-3F |  | GCGAATCTTAATTACCGCTTCCTGC |  | | nhx5-3R |  | CGACAAGAAGATGATAACCCT |
|  | nhx5-4F |  | GCCTTACCACTCGTCTGCTGT |  | | nhx5-4R |  | CCCTCCAGCGATAATGAATAA |
|  | nhx5-5F |  | GCTTGTTGATGCGTGTTGGAAT |  | | nhx5-5R |  | TTTGCTGCTGCTTTCTATCAC |
| *NHX6* | nhx6-1F |  | AGAAAGAATGATGGCTTACGG |  | | nhx6-1R |  | TGTTGATTCCAATACCGCATG |
|  | nhx6-2F |  | TGATTCACTGTCTGTCATTTCCCTT |  | | nhx6-2R |  | AAGGTTTGACAATACATTTGAGC |
|  | nhx6-3F |  | GAACCTTTTCAAGTATGCTGGTT |  | | nhx6-3R |  | AGTATCCAACTGAAAGATGAACTCG |
|  | nhx6-4F |  | GGCTCCCATTCACTTGAAATT |  | | nhx6-4R |  | AGGCTAATGGACCTAACTTCT |
|  | nhx6-5F |  | CAAGAACAAAGGGCTAGTTTAT |  | | nhx6-5R |  | AGGGAGAACCCTGGATCAAGT |
| *NhaD1* | nhad1-1F |  | GTGGCTAGGCAAGTGGAAAGT |  | | nhad1-1R |  | CATAACTATTGGTCCTTCGAGTAAC |
|  | nhad1-2F |  | AGACATTATTTGATGAGGAGGCA |  | | nhad1-2R |  | TTTAGGCTCAGTTGTCTGTCT |
|  | nhad1-3F |  | AGACAGACAACTGAGCCTAAA |  | | nhad1-3R |  | AACCAGTCTATTTCAGGGTAA |
|  | nhad1-4F |  | TGTATTCCCTAGTATAGAGTTGG |  | | nhad1-4R |  | ATAACCAAGAAAACCACAACC |
|  | nhad1-5F |  | GTGCTTAGAATTTTTCCCTCGTT |  | | nhad1-5R |  | GGCAAGCATAATGTGATCCGTC |
| *NhaD2* | nhad2-1F |  | CTGTCGATAAGCCATTTCCAT |  | | nhad2-1R |  | AACCTGTCCTAATATGTTCAGATGC |
|  | nhad2-2F |  | GTTACCAACACTTGCATCTGAACAT |  | | nhad2-2R |  | TGCCTCAAGTTAACACATCCTAG |
|  | nhad2-3F |  | AACTTTGCCTGAACCACCTTT |  | | nhad2-3R |  | GTCATGCACTCTTTGTTATTT |
|  | nhad2-4F |  | GGAGAAATCGGTCCCTTGT |  | | nhad2-4R |  | AGAGGGTACAAGCAAGTCC |
|  | nhad2-5F |  | GTGTCTCAGGACCCGACTTCT |  | | nhad2-5R |  | GTGTCTCAGGACCCGACTTCT |
|  | nhad2-6F |  | GTGTTATGTCTAGGTTTGGGTT |  | | nhad2-6R |  | GCAATTTTGGAGGAGCACCGTCT |
| *SOS1* | sos1-1F |  | CAGTTTCAGTGGAGGAGCAAG |  | | sos1-1R |  | CCTAAACGAAAGCAACCAACT |
|  | sos1-2F |  | CCTTCGACACCTGATTGTAGTT |  | | sos1-2R |  | CACAGACAGAGAAACAGAGGAGG |
|  | sos1-3F |  | GAGGCAGATTTGGTGTATGAT |  | | sos1-3R |  | GCGAGGTAAATGGTTTCTAATG |
|  | sos1-4F |  | CATGTGAATGGAACTTTCCAAC |  | | sos1-4R |  | TGCAAAACCTTCTCTTCTACA |
|  | sos1-5F |  | CACGAGGTGCCACATTAGTTAG |  | | sos1-5R |  | CAAAATCACAAACATCACTGA |
|  | sos1-6F |  | TCCATTTCTATTCCCTTCCTG |  | | sos1-6R |  | TAACATACATCCCAGCAGACA |
|  | sos1-7F |  | GCTTGTTATGTTATCTAGCGGATGT |  | | sos1-7R |  | CCTCTGTTAAGATTGGTGTAC |
|  | sos1-8F |  | AAATGTACTCTCGGTGGCTTCC |  | | sos1-8R |  | GGCAACTAGACACTGTGGATTGG |
|  | sos1-9F |  | TTCAGCACATTTCTCCTCCAT |  | | sos1-9R |  | GCTATTGGGTTTGGGTTGTGA |
|  | sos1-10F |  | TTCCTACATCAACATGAGCAC |  | | sos1-10R |  | TAGCATGGAGGCACAATGAACT |
|  | sos1-13F |  | GAGGGCTTCTTTTATATT |  | | sos1-13R |  | ACATCTTGTTTACAACGGTTCC |
|  | sos1-14F |  | TCACTGCTATTGTTGGTT |  | | sos1-14R |  | CTAAGAAGCATGATGGAACG |
| *SOS1B* | sos2-1F |  | CACTGTCTTATTATCCGTTTAGACC |  | | sos1-1R |  | ACAAACCAAATAACAAGGAGG |
|  | sos2-5F |  | AACCACCTATGTCCTCTAATC |  | | sos1-5R |  | TCATGGGAACTATGAGGAAAC |
|  | sos2-6F |  | TTGTGACATCACATGTCCATGCACT |  | | sos1-6R |  | AGCAGAACAAATGTAACTCCC |
|  | sos2-7F |  | AAAGACACCAAAGAGGCACTC |  | | sos1-7R |  | AGACCCAGACCAACCACAGAA |
|  | sos2-9F |  | GGGTTGGGTAGAAGAATGTTG |  | | sos1-9R |  | AAGCATTGTTACGAAGGGAGA |
|  | sos2-11F |  | CCTCCATCCATGAAATTACTC |  | | sos1-11R |  | CATGATAGGTTCTTGGGTTGG |
|  | sos2-12F |  | CTTAACCATCATGTAAACAGG |  | | sos1-12R |  | CCGAAAGCCACAGAAATTCAATAAT |

***Table S2*** *Summary statistics for each of 10 Na^+^/H^+^ antiporter loci in 17 populations of P. euphratica and* P. pruinosa.

| Species | Locus | N | L | S | θ_wt_ | π_t_ | L_sil_ | S_sil_ | θ_sil_ | π_sil_ | N_h_ | H_d_ | R_m_ | Zns | F_ST_ |
| --- | --- | --- | --- | --- | --- | --- | --- | --- | --- | --- | --- | --- | --- | --- | --- |
| *P.euphratica* | *SOS1* | 48 | 8858 | 34 | 0.00147 | 0.00116 | 6244.52 | 27 | 0.00097 | 0.00125 | 31 | 0.981 | 8 | 0.1070 | 0.54446^***^ |
|  | *SOS1B* | 48 | 5214 | 71 | 0.00307 | 0.00278 | 3875.74 | 59 | 0.00343 | 0.00312 | 21 | 0.948 | 12 | 0.1416 | 0.51299^***^ |
|  | *NHX1* | 84 | 4368 | 54 | 0.00252 | 0.00254 | 3405.83 | 52 | 0.00305 | 0.00311 | 63 | 0.985 | 18 | 0.0726 | 0.4979^***^ |
|  | *NHX2* | 88 | 4878 | 99 | 0.00414 | 0.00503 | 3629.02 | 98 | 0.00535 | 0.00657 | 49 | 0.967 | 49 | 0.967 | 0.49426^***^ |
|  | *NHX3* | 88 | 4023 | 50 | 0.00246 | 0.00121 | 2779.46 | 47 | 0.00335 | 0.00169 | 50 | 0.955 | 12 | 0.0413 | 0.31551^***^ |
|  | *NHX4* | 88 | 4251 | 12 | 0.00056 | 0.00065 | 3043.4 | 11 | 0.00072 | 0.00084 | 13 | 0.853 | 3 | 0.082 | 0.2534^***^ |
|  | *NHX5* | 88 | 3890 | 27 | 0.00137 | 0.00114 | 2657.44 | 27 | 0.00201 | 0.00167 | 34 | 0.790 | 7 | 0.0749 | 0.28276^***^ |
|  | *NHX6* | 88 | 5033 | 28 | 0.00110 | 0.00136 | 3853.30 | 27 | 0.00139 | 0.00175 | 29 | 0.927 | 8 | 0.1049 | 0.30589^***^ |
|  | *NhaD1* | 88 | 4342 | 42 | 0.00196 | 0.00229 | 3197.61 | 40 | na | 0.00303 | 44 | 0.972 | 12 | 0.0992 | 0.42892^***^ |
|  | *NhaD2* | 88 | 5468 | 132 | 0.00482 | 0.00881 | 4157.08 | 122 | na | 0.01066 | 24 | 0.911 | 10 | 0.5716 | 0.43395^***^ |
| *P.pruinosa* | *SOS1* | 38 | 8807 | 70 | 0.00192 | 0.00153 | 6196.62 | 64 | 0.00249 | 0.00203 | 28 | 0.984 | 8 | 0.1528 | 0.36016^***^ |
|  | *SOS1B* | 38 | 5236 | 48 | 0.00218 | 0.00189 | 3897.87 | 43 | 0.00263 | 0.00230 | 20 | 0.945 | 7 | 0.1727 | 0.16986^***^ |
|  | *NHX1* | 58 | 4364 | 73 | 0.00366 | 0.00413 | 3401.87 | 72 | 0.00457 | 0.00515 | 52 | 0.996 | 19 | 0.0893 | 0.12568^***^ |
|  | *NHX2* | 56 | 4881 | 78 | 0.00366 | 0.00604 | 3633.39 | 80 | 0.00479 | 0.00797 | 43 | 0.988 | 21 | 0.1943 | 0.4629^***^ |
|  | *NHX3* | 60 | 4019 | 75 | 0.00406 | 0.00366 | 2775.50 | 76 | 0.00587 | 0.00530 | 43 | 0.968 | 19 | 0.1428 | 0.19137^***^ |
|  | *NHX4* | 62 | 4251 | 47 | 0.00242 | 0.00142 | 3020.19 | 46 | 0.00324 | 0.00169 | 25 | 0.906 | 8 | 0.1983 | 0.12365^***^ |
|  | *NHX5* | 64 | 3892 | 51 | 0.00288 | 0.00383 | 2659.19 | 49 | 0.00390 | 0.00498 | 42 | 0.983 | 17 | 0.1494 | 0.25741^***^ |
|  | *NHX6* | 52 | 5012 | 51 | 0.00225 | 0.00322 | 3734.42 | 47 | 0.00271 | 0.00407 | 40 | 0.988 | 20 | 0.1294 | 0.15777^***^ |
|  | *NhaD1* | 64 | 4390 | 19 | 0.00092 | 0.00098 | 3245.11 | 17 | 0.00111 | 0.00118 | 30 | 0.958 | 7 | 0.0863 | 0.145^***^ |
|  | *NhaD2* | 54 | 4666 | 121 | 0.00588 | 0.00727 | 3354.38 | 116 | 0.00759 | 0.00940 | 46 | 0.993 | 22 | 0.0942 | 0.23656^***^ |

*, **P < 0.05** **, **P < 0.01,** ^***^, **P < 0.001**

N, sample size; L, length in base pairs; S, number of segregating sizes; π, nucleotide diversity (Nei 1987; Nei & Li, 1979); θ, Watterson’s parameter (Watterson, 1975); Nh, number of haplotypes; Hd, Haplotype (gene) diversity; Rm, minimum number of recombinant events; Zns, Linkage Disequilibrium (Kelly 1997), F_ST_, Population differentiation.

**Table S3** Gene-specific primers used for 16 reference loci and genomic locations.

| Locus | Primers |  | Locations in *P.euphratica* genome |
| --- | --- | --- | --- |
| *174078* | AATCGTGCCCAATCTACGAC  CGCTTCGACGGTCTTCTGTTC |  | scaffold_310: 190941-195129 |
| *195487* | AGATTGTTGTTCCTGCCGAT  CCATGGCCGTCCTAGAAATGT |  | scaffold_91: 237705-239274 |
| *230239* | GCCAAAGCTCGTGTAGGAAG  CGCCTCCTCGGTATTCTGTGA |  | scaffold_3615: 6579-9180 |
| *282601* | CAATTCATCATCGTCCGC  CCTCGCCAATATCACAAACGA |  | scaffold_46: 1634977-1637031 |
| *423802* | ACCATCGACCTTTCCATCAC  CACGTGCAAGATTTGCATCAC |  | scaffold_8: 458602-459387 |
| *424060* | TTCGGTTTCATCCTACGGAC CGGATTGGTGAGATTGGCA |  | scaffold_4: 562749-564532 |
| *554813* | GCCTTTGGTCTGGTCTCTTG  CAGCAAGGGCATTTGCATAAA |  | scaffold_8: 1704246-1709797 |
| *554898* | AGCAAGTTCTCAGGCAGGAA  CGCCAATCATCCAAACTTCCA |  | scaffold_8: 1063994-1072763 |
| *560952* | GTCATAACAAGCTTTCCGGG  CCCATGCTTTCCTTTTGAAGC |  | scaffold_46: 2355468-2358434 |
| *588180* | CACTCATGGGTGAAAACGTG  CCTCTTTCTTTCCGCGATCAG |  | scaffold_246: 141275-143051 |
| *652105* | GGAGGATTTTCAGTTCACGC  CCGTCACAGCCTCACAAGCTA |  | scaffold_236: 19142-24452 |
| *678140* | TTAAGTCCCAGGGGAAGAGC CCACACTCCATTGCACCTTTG |  | scaffold_312: 230088-230943 |
| *706088* | ATCTGCCTACCATCTGTGCC CAGCCTCAACAGCCTTCAAAA |  | scaffold_42: 591882-606770 |
| *751129* | CATTTAGCTCTGGCGCTTTC CCAGCTTGCCTTCCAATTCA |  | scaffold_7: 606081-608016 |
| *781842* | GCTTTCATCCGCATAGTGGT  CATGGATACCCTCCATAGCCC |  | scaffold_332: 59874-65603 |
| *816152* | GGTGCTGGTTTCACCAGATT  CATTTCCAGCATTTGCTCTGC |  | scaffold_30: 1301587-1305660 |

Olson MS, Robertson AL, Takebayashi N et al. (2010). Nucleotide diversity and linkage disequilibrium in balsam poplar (*Populus balsamifera*). *New Phytologist,* 186, 526-536.

Ma T, Wang JY, Zhou GK et al. (2013). Genomic insights into salt adaptation in a desert poplar. *Nature Communications*, 4, 2797.

**Table S4** Summary statistics for 16 reference loci in 17 populations of *P. euphratica* and *P. pruinosa.*

| Species | Locus | N | L | S | θ_wt_ | π_t_ | Lsil | Ssil | θ_sil_ | π_sil_ | N_h_ | H_d_ | R_m_ | | Zns | F_ST_ |
| --- | --- | --- | --- | --- | --- | --- | --- | --- | --- | --- | --- | --- | --- | --- | --- | --- |
| *P.euphratica* | *174078* | 84 | 457 | 5 | 0.00219 | 0.00108 | 375 | 5 | 0.00267 | 0.00132 | 5 | 0.336 | 0 | 0.1023 | | 0.23124^***^ |
|  | *195487* | 86 | 599 | 17 | 0.00598 | 0.00561 | 329.40 | 13 | 0.00785 | 0.00656 | 14 | 0.805 | 4 | 0.1600 | | 0.30653^***^ |
|  | 280517 | 82 | 567 | 6 | 0.00213 | 0.00251 | 129.43 | 2 | 0.00310 | 0.00541 | 8 | 0.743 | 1 | 0.0795 | | 0.11917^**^ |
|  | *282601* | 86 | 582 | 0 | 0 | 0 | 139.83 | 0 | 0 | 0 | 1 | 0 | na | na | | 0.0000 |
|  | *423802* | 84 | 411 | 5 | 0.00243 | 0.00130 | 97.64 | 2 | 0.00410 | 0.00312 | 5 | 0.445 | 0 | 0.1413 | | 0.2343^***^ |
|  | *424060* | 64 | 671 | 3 | 0.000954 | 0.00077 | 393.20 | 2 | 0.00108 | 0.00102 | 5 | 0.439 | 1 | 0.0191 | | 0.38238^***^ |
|  | *554813* | 84 | 553 | 5 | 0.00181 | 0.00127 | 130.03 | 3 | 0.00461 | 0.00275 | 7 | 0.525 | 2 | 0.0307 | | 0.37079^***^ |
|  | *554898* | 84 | 669 | 8 | 0.00209 | 0.00190 | 568.50 | 2 | 0.00246 | 0.00223 | 8 | 0.658 | 1 | 0.1876 | | 0.31338^***^ |
|  | *560952* | 88 | 580 | 8 | 0.00273 | 0.00119 | 144.27 | 4 | 0.00549 | 0.00265 | 6 | 0.424 | 0 | 0.1562 | | 0.26029^***^ |
|  | *583031* | 78 | 673 | 7 | 0.00211 | 0.00307 | 549.67 | 7 | 0.00258 | 0.00376 | 10 | 0.761 | 2 | 0.1916 | | 0.23253^***^ |
|  | *588180* | 80 | 563 | 7 | 0.00251 | 0.00368 | 208.67 | 6 | 0.00581 | 0.00924 | 8 | 0.666 | 0 | 0.2370 | | 0.17158^**^ |
|  | *593178* | 78 | 631 | 6 | 0.00193 | 0.00179 | 181.15 | 5 | 0.00560 | 0.00571 | 3 | 0.293 | 0 | 0.6690 | | 0.31278^***^ |
|  | *652105* | 82 | 580 | 9 | 0.00312 | 0.00157 | 453.83 | 9 | 0.00398 | 0.00200 | 5 | 0.584 | 0 | 0.4205 | | 0.41834^***^ |
|  | *678140* | 88 | 498 | 4 | 0.00159 | 0.00249 | 374.72 | 0 | 0 | 0 | 5 | 0.766 | 0 | 0.1157 | | 0.39987^***^ |
|  | *706088* | 84 | 509 | 15 | 0.00589 | 0.00490 | 397.00 | 15 | 0.00755 | 0.00628 | 12 | 0.646 | 3 | 0.1647 | | 0.22785^***^ |
|  | *816152* | 86 | 618 | 7 | 0.00225 | 0.00172 | 405.49 | 6 | 0.00294 | 0.00251 | 4 | 0.675 | 0 | 0.4960 | | 0.22027^***^ |
| *P. pruinosa* | *174078* | 52 | 457 | 13 | 0.00630 | 0.00300 | 374.36 | 12 | 0.00709 | 0.00355 | 7 | 0.400 | 2 | 0.5527 | | 0.17371^*^ |
|  | *195487* | 52 | 599 | 11 | 0.00443 | 0.00397 | 331.18 | 9 | 0.00601 | 0.00619 | 8 | 0.830 | 1 | 0.2377 | | 0.22441^***^ |
|  | *230239* | 54 | 639 | 13 | 0.00446 | 0.00404 | 135.75 | 6 | 0.00970 | 0.00746 | 10 | 0.608 | 3 | 0.2194 | | 0.15143^*^ |
|  | *280517* | 46 | 567 | 7 | 0.00281 | 0.00160 | 129.50 | 6 | 0.01054 | 0.00523 | 5 | 0.316 | 0 | 0.2459 | | 0.05304 |
|  | *282601* | 40 | 582 | 3 | 0.00121 | 0.00102 | 139.75 | 2 | 0.00336 | 0.00263 | 3 | 0.347 | 0 | 0.3411 | | 0.13504 |
|  | *423802* | 52 | 411 | 3 | 0.00162 | 0.00201 | 97.94 | 2 | 0.00452 | 0.00522 | 4 | 0.565 | 0 | 0.1432 | | 0.08027 |
|  | *424060* | 42 | 671 | 6 | 0.00242 | 0.00211 | 395.17 | 7 | 0.00412 | 0.00358 | 7 | 0.735 | 0 | 0.1269 | | 0.05676 |
|  | *554813* | 60 | 553 | 5 | 0.00194 | 0.00207 | 129.47 | 1 | 0.00106 | 0.00235 | 4 | 0.439 | 1 | 0.6037 | | 0.13118^*^ |
|  | *554898* | 52 | 669 | 17 | 0.00562 | 0.00771 | 568.50 | 17 | 0.00662 | 0.00907 | 10 | 0.824 | 1 | 0.3129 | | 0.15904^**^ |
|  | *560952* | 54 | 580 | 10 | 0.00378 | 0.00374 | 144.44 | 6 | 0.00912 | 0.00724 | 9 | 0.803 | 0 | 0.0959 | | 0.12756^**^ |
|  | *588180* | 48 | 563 | 9 | 0.00360 | 0.00490 | 208.67 | 7 | 0.00756 | 0.01207 | 11 | 0.863 | 2 | 0.1347 | | 0.02361 |
|  | *652105* | 54 | 580 | 4 | 0.00151 | 0.00114 | 453.83 | 4 | 0.00193 | 0.00145 | 4 | 0.405 | 0 | 0.2495 | | 0.10824^*^ |
|  | *678140* | 52 | 498 | 12 | 0.00533 | 0.00653 | 375.02 | 9 | 0.00531 | 0.00702 | 10 | 0.764 | 3 | 0.1837 | | 0.12005^**^ |
|  | *706088* | 60 | 509 | 12 | 0.00506 | 0.00482 | 397.00 | 12 | 0.00648 | 0.00618 | 8 | 0.746 | 1 | 0.2512 | | 0.33667^***^ |
|  | *751129* | 34 | 600 | 3 | 0.00122 | 0.00081 | 140.50 | 2 | 0.00348 | 0.00304 | 3 | 0.266 | 0 | 0.3360 | | 0.38123^*^ |
|  | *816152* | 50 | 618 | 8 | 0.00289 | 0.00185 | 405.17 | 6 | 0.00331 | 0.00234 | 4 | 0.474 | 0 | 0.5453 | | 0.13402^*^ |

*, **P < 0.05** **, **P < 0.01,** ^***^, **P < 0.001**

N, sample size; L, length in base pairs; S, number of segregating sizes; π, nucleotide diversity (Nei 1987; Nei & Li, 1979); θ, Watterson’s parameter (Watterson, 1975); Nh, number of haplotypes; Hd, Haplotype (gene) diversity; Rm, minimum number of recombinant events; Zns, Linkage Disequilibrium (Kelly 1997), F_ST_, Population differentiation
